# Trend analysis and prediction of the incidence and mortality of CKD in China and the US

**DOI:** 10.1186/s12882-024-03518-w

**Published:** 2024-03-01

**Authors:** Wenpeng Zhu, Mengqi Han, Yuxin Wang, Guoping Wang

**Affiliations:** School of Public Health, Wannan Medical College, Wuhu, Anhui Province China

**Keywords:** CKD, Incidence, Mortality, Joinpoint, Age-period-cohort, Prediction

## Abstract

**Background:**

Currently, limited research is available on the comparative analysis of chronic kidney disease (CKD) incidence and mortality rates between China and the United States. This study aimed to explore the trends in CKD incidence and mortality rates in both countries, as well as make some future predictions.

**Methods:**

The data on CKD incidence and mortality in China and the US from 1990 to 2019 were derived from the 2019 Global Burden of Disease database. A Joinpoint regression model was used to analyze temporal trends in CKD incidence and mortality. An age-period-cohort model was used to assess the effects of age, period, and birth cohort on CKD risk and forecast the age-standardized incidence rate (ASIR) and age-standardized mortality rate (ASMR) of CKD in China and the US over the next 15 years.

**Results:**

CKD incidence in China and the US showed an upward trend. Its mortality rate showed a downward trend in China but an upward one in the US. The relative risk (RR) of CKD incidence and mortality increases with age. The RR of CKD incidence in the 0–5 age group exceeds that in the 5–55 age group, and the RR for mortality surpasses that in the 5–35 age group. Over time, the RR of CKD incidence has gradually increased in China and the US. Individuals born in later birth cohorts had a lower RR of CKD incidence and mortality. The ASIR of CKD may increase in both China and the US, whereas its ASMR may decline over the next 15 years.

**Conclusion:**

Screening measures should be strengthened among populations at high risk of CKD; prenatal examinations of pregnant women should be emphasized to reduce CKD incidence in newborns. It is imperative to increase health education and encourage individuals to adopt healthy lifestyles.

**Supplementary Information:**

The online version contains supplementary material available at 10.1186/s12882-024-03518-w.

## Background

Chronic kidney disease (CKD) is characterized by kidney damage or a glomerular filtration rate (GFR) of ≤ 60 mL/min/1.73 m^2^ for > 3 consecutive months. An expanded definition of CKD also includes a one-time urine albumin-to-creatinine ratio ≥ 30 mg/g [1]. CKD is a major global public health concern. In recent years, its high incidence rate, hospitalization rate, and associated costs, along with its poor prognosis, have had a substantial impact on the quality of life of patients with CKD and have placed increased burdens on their families. The disability-adjusted life years associated with CKD per thousand people is 17.22 years, surpassing even that of cancer [2]. CKD profoundly affects health, significantly increasing the risks of end-stage renal disease, cardiovascular disease, and death [3, 4]. The later stages of CKD are characterized by high rates of complications and mortality. Although cardiovascular and cerebrovascular diseases have received considerable attention, the concurrent presence of kidney disease and the importance of CKD tend to be overlooked. However, CKD plays a crucial role in the patient’s quality of life and the progression and longevity of other diseases, such as stroke, peripheral vascular disease, and non-alcoholic fatty liver disease. Research indicates that there are 697.5 million patients with CKD worldwide, accounting for 9.1% of the global population [5]. From 1990 to 2017, the global mortality rate of CKD increased by 41.5% [6]. CKD was identified as the 12th leading cause of death worldwide in 2017 and is predicted to become the fifth leading cause of mortality worldwide by 2040 [7].

A cross-sectional analysis involving 176,874 participants revealed that CKD prevalence in China was 8.2%, with an estimated population of over 20 million patients with CKD between 1982 and 2018 [8]. CKD prevalence in the US is 6.9% and has remained relatively stable in recent years [9]. However, few studies have compared the incidence and mortality of CKD in China and the US. By comparing the trends in the incidence and mortality of CKD in the two countries, we can understand the prevalence of the disease from a global perspective. This helps to identify and understand the common risk factors, disease patterns, and trends of CKD and provides a reference for global health policies and interventions. Moreover, China and the US are both countries that span a large geographic area and have populations of over 300 million. Both countries are experiencing population aging and share similar lifestyle factors, including poor dietary habits, lack of exercise, smoking, and alcohol consumption. At the same time, a range of chronic disease prevention and treatment policies have been implemented. Data on CKD incidence and mortality in both countries were obtained from the Global Burden of Disease (GBD) database, from similar sources, and with the same diagnostic criteria, which made the epidemiologic studies of CKD in the two countries more comparable [10].. Therefore, this study aimed to analyze the trends in CKD incidence and mortality in China from 1990 to 2019, compare them with the corresponding trends in the US, and predict future changes in CKD over the next 15 years. The findings of this study will hopefully provide a scientific basis for the development of future prevention and treatment strategies for CKD.

## Methods

### Data sources

Data were obtained from the 2019 GBD, released by the Institute for Health Metrics and Evaluation at the University of Washington, USA (https://ghdx.healthdata.org/gbd-2019). The dataset was compiled by collecting all available data, standardizing it, and then using models to estimate the burden of 369 diseases or injuries across 204 countries and territories worldwide from 1990 to 2019. The countries and territories were classified based on their social demographic index (SDI) into low, low-middle, middle, high-middle, and high SDI categories. The burden of diseases was further analyzed by age and sex [11]. The data on CKD incidence and mortality in China is primarily sourced from the National Disease Surveillance System, the Resident Death Registration Information System, and the National Maternal and Child Health Surveillance System, besides published literature and reports. Similarly, in the US, data on CKD incidence and mortality is gathered from a variety of sources, including the United States Census Bureau, the National Center for Health Statistics, the National Death Registration System, and the Chronic Disease Surveillance System, alongside clinical research and epidemiological surveys [12].. In this study, we extracted the age-standardized incidence rate (ASIR) and age-standardized mortality rate (ASMR) of Chinese and American residents in all age groups from 1990 to 2019.

### Methods

Joinpoint regression software 4.9.0.0 was used to calculate the annual percentage change (APC) and average annual percentage change (AAPC) of CKD incidence and mortality in different countries and across the sexes. An APC of > 0 indicated an upward trend, and an APC of < 0 indicated a downward one. If APC = AAPC, it meant that there was no significant change in the rate in that group, and the rate was monotonically increasing or decreasing [13]. The significance level α was 0.05 (*P* < 0.05), and this was the cutoff for statistical significance.

The age-period-cohort model can be used to accurately estimate changes in diseases affected by age, period, and cohort factors [14]. To resolve the linear relationships among age, period, and cohort, the Intrinsic Estimator (IE) method proposed by Yang and Fu was applied [15, 16]. Based on the estimation function method, they proposed the intrinsic estimator based on the estimation function and matrix singular value decomposition. Compared with the traditional generalized linear model, which assumes that two or more coefficients of a parameter vector are equal, the IE limits the geometric orientation of the parameter vector in the parameter space. Age was divided into one age group every 5 years, the period was divided into 5 years, and the birth cohort was divided into 5 years. The Stata 17.0 IE package was used to analyze the effect coefficient and relative risk (RR) of the age, period, and cohort of onset and death due to CKD, with a positive effect coefficient (i.e., RR) indicating increased risk and a negative one indicating reduced risk. The basic expression for the age-period-cohort model takes the form:

$$ \mathbf{ln}\left({\varvec{Y}}_{\varvec{a}\varvec{b}\varvec{c}}\right)=\varvec{\mu }+{\varvec{\alpha }}_{\varvec{a}}+{\varvec{\beta }}_{\varvec{b}}+{\varvec{\gamma }}_{\varvec{c}}$$

where **ln (*Yabc*)** is the natural logarithm of CKD mortality rate or incidence rate; ***µ*** is the intercept that indicates the reference level of disease risk of age, period, and cohort parameters; ***α***_***a***_ is the age effect of age group a, a = 1,2,…; ***β***_***b***_ is the period effect of period b, b = 1,2,…; ***γ***_***c***_ is the cohort effect of the **c**th cohort, ***γ***_***c***_ = b– a + n, n is the number of age groups. The significance level α was 0.05 (*P* < 0.05), which was considered statistically significant.

The BAPC package for R 4.2.2 software was used to predict the mortality and incidence of a given disease. This approach allowed us to provide insights into potential future scenarios regarding the CKD burden in China and the US. The model can be expressed as follows:

$$ {\varvec{\eta }}_{\varvec{i}\varvec{j}}=\mathbf{log}\left({\varvec{\lambda }}_{\varvec{i}\varvec{j}}\right)=\hspace{0.17em}\varvec{\mu }\hspace{0.17em}+\hspace{0.17em}{\varvec{\alpha }}_{\varvec{i}} +{\varvec{\beta }}_{\varvec{j}}\hspace{0.17em} +\hspace{0.17em}{\varvec{\gamma }}_{\varvec{k}}$$

where ***λ*** indicates incidence or mortality rate;$\varvec{\eta }$ indicates linear predictors; ***µ*** represents the intercept, and ***α***_***i***_, ***β***_***j***_, and ***γ***_***k***_ represent age, period, and cohort effects, respectively. The ***i*** (1 ≤ ***i*** ≤ ***I***) represents the age group of period j (1 ≤ ***j*** ≤ ***J***); k represents the cohort index, ***k*** = ***M*** (***I***- ***i***) + ***j***, depending on the age, period, and age group width and period interval; and M indicates that the age group interval is M times wider than the period interval. For example, for the 5-year age group, M is 5. In the BAPC model, all unknown parameters are considered to be random and have appropriate prior distributions. The model applies a second-order random walk model to smooth the prior incidence and mortality rates for age, period, and cohort effects. The nested Laplace approximation method is used to approximate the marginal posterior distributions and predict the posterior incidence and mortality rates [17]. The significance level α was 0.05 (*P* < 0.05), which was considered statistically significant.

## Results

### Analysis of CKD incidence trend in China and the US

**Fig. 1:**
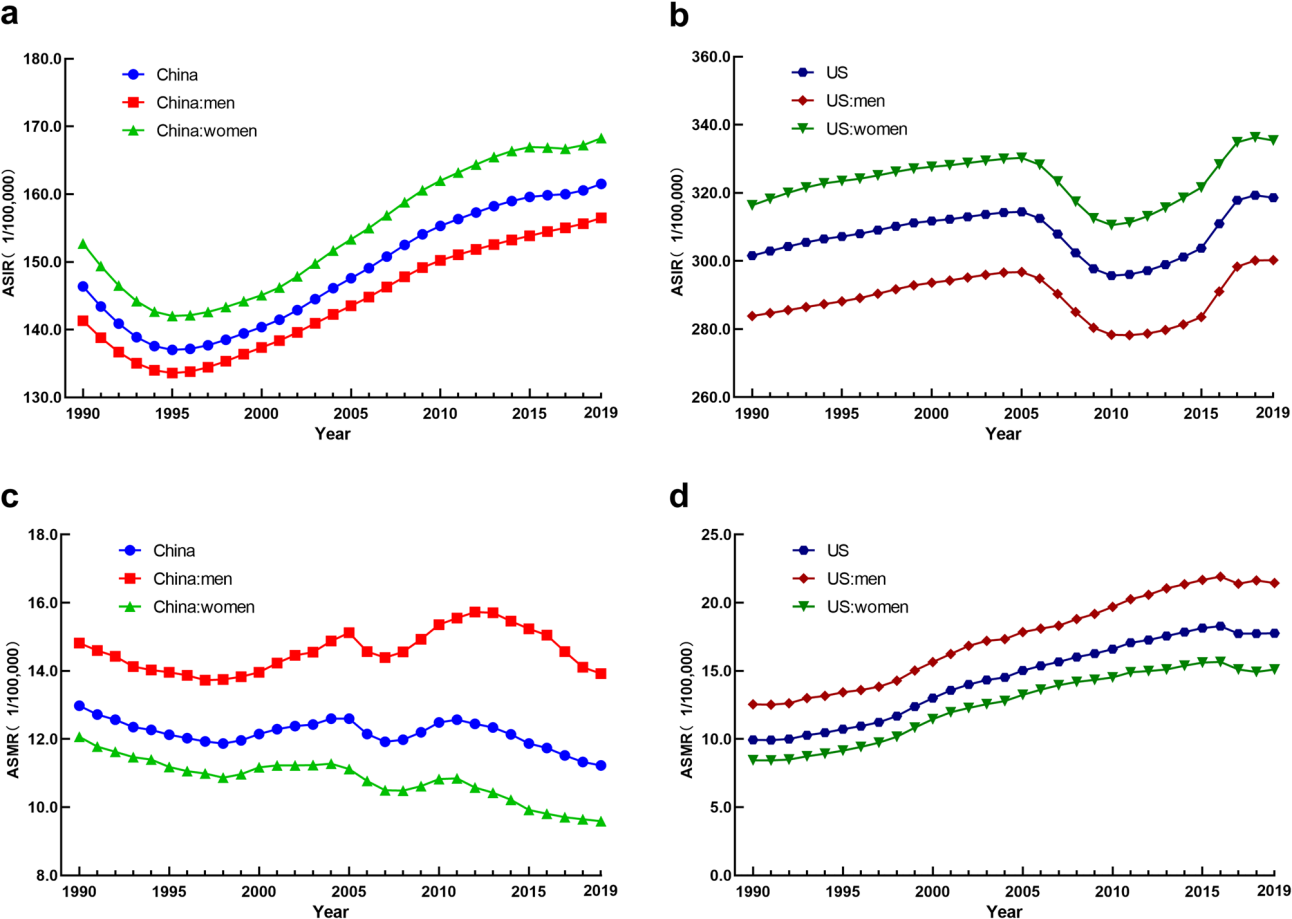
Analysis of CKD incidence and mortality trend (**a**: China of incidence, **b**: the US of incidence, **c**: China of mortality, **d**: the US of mortality)

From 1990 to 2019, the ASIR of CKD in Chinese residents, men, and women showed an overall upward trend (AAPC = 0.30, 0.29, and 0.29, respectively; *P* < 0.05), with a downward trend from 1990 to 1996 and another upward trend after 1996. The ASIR of CKD in US residents, males, and females showed an overall upward trend (AAPC = 0.18, 0.20, and 0.20, respectively; *P* < 0.05), with a downward trend from 2005 to 2010 and an upward trend in other years. The ASIR for CKD in the US was higher than that in China, and the ASIR for CKD in women was higher than that in men (Fig. 1; Table 1).

**Table 1:** AAPC of CKD, ASIR, and ASMR in China and the US

Group	ASIR				ASMR		
	APPC	95~CI	P		APPC	95~;CI	P
**China**	0.30	0.26~0.34	<0.01		-0.59	-0.73~-0.44	<0.01
**China:men**	0.29	0.26~0.33	<0.01		-0.30	-0.54~-0.06	0.01
**China:women**	0.29	0.24~0.35	<0.01		-0.89	-1.09~-0.69	<0.01
**US**	0.18	0.11~0.24	<0.01		2.04	1.83~2.26	<0.01
**US:men**	0.20	0.14~0.25	<0.01		1.94	1.75~2.13	<0.01
**US:women**	0.20	0.16~0.25	<0.01		2.04	1.76~2.31	<0.01

### Analysis of CKD mortality trend in China and the US

The ASMR of CKD in China showed a downward trend (AAPC = -0.59, *P* < 0.05), as well as a downward trend in both men and women (AAPC = -0.30 and − 0.89, respectively; *P* < 0.05). The ASMR for CKD in the US showed an upward trend (AAPC = 2.04, *P* < 0.05), as well as in both men and women (AAPC = 1.94 and 2.04, respectively; *P* < 0.05). We also found that the ASMR of CKD in men was higher than that in women (Fig. 1; Table 1).

### Age-period-cohort analysis of CKD incidence and mortality in China and the US

#### Age-period-cohort analysis of CKD incidence

The age effect showed that CKD in China and the US had a higher RR of incidence in the 0–5 age group (RR = 1.99, 1.43) than they did in other age groups and then remained low (RR_min_= 0.16, 0.12). After the age of 45 years, the RR of incidence increased rapidly, was higher in the US than in China, and increased to its highest level in the 75–80 age group (RR = 7.25, 9.06).

The period effect showed that the RR of CKD incidence increased over time in both countries. The RR of CKD incidence in China and the US was the lowest in 1990–1994 (RR = 0.74, 0.76) and the highest in 2015–2019 (RR = 1.39, 1.36).

**Fig. 2:**
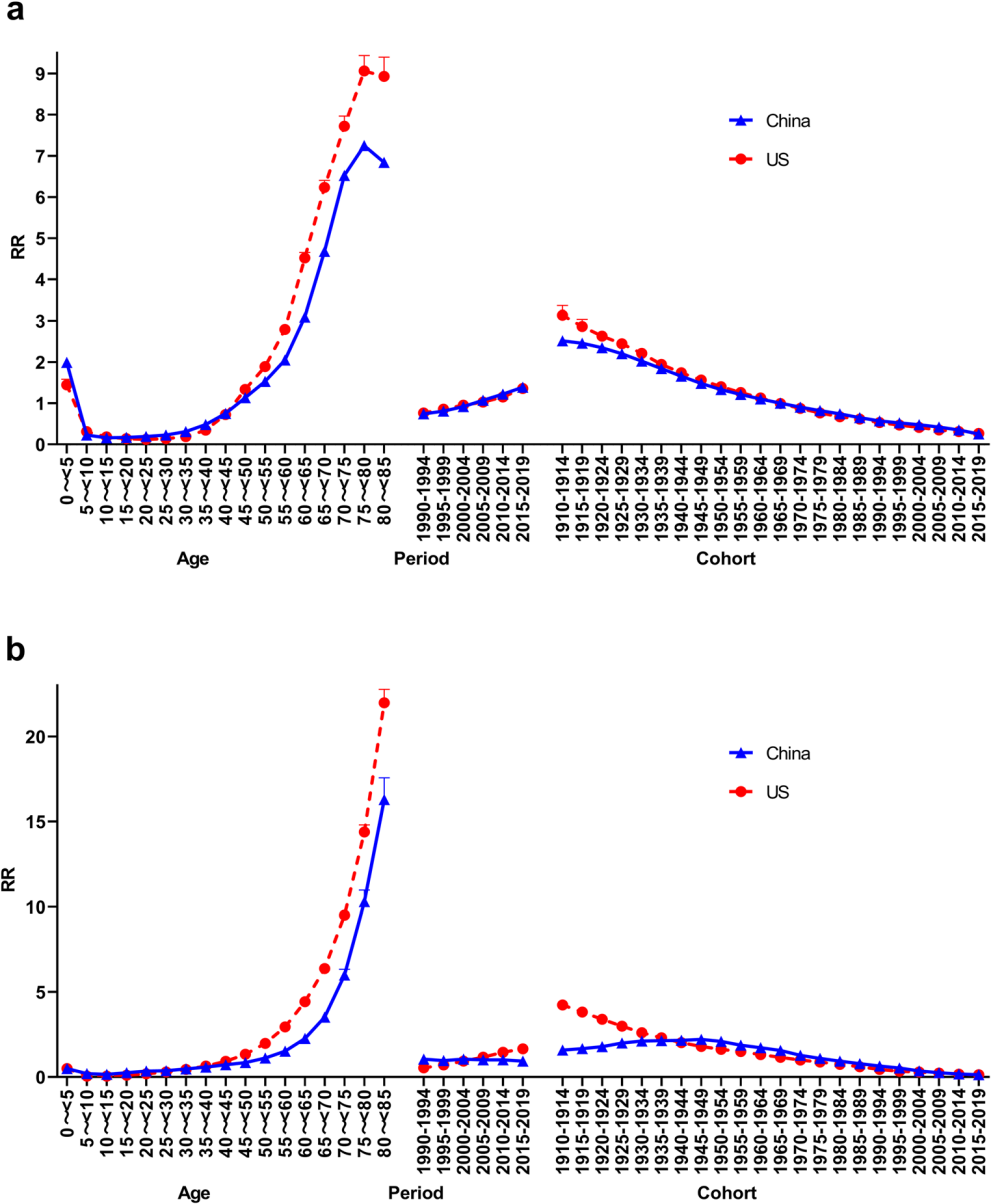
Age-period-cohort analysis of CKD incidence and mortality in China and the US (**a**: incidence, **b**: mortality)

The cohort effect showed that the RR of CKD incidence decreased with the progression of the cohort in both countries. The RR of CKD incidence in China and the US was the highest in the cohort born in 1910–1915 (RR = 2.51, 3.13) and the lowest in the 2015–2019 birth cohort (RR = 0.25, 0.26)(Fig. 2).

### Age-period-cohort analysis of CKD mortality

The age effect showed that the RR of mortality increased overall with age in both China and the US. The RR of mortality was the lowest in the 10–15 age group in both countries (RR = 0.15, 0.04). After the age of 50, the RR of CKD mortality increased rapidly, more so in the US than in China, and it increased the most in the 80–85 age group (RR = 16.26, 21.98).

The period effect showed that the RR of CKD mortality in China did not change significantly over the years, with its highest RR = 1.05 and its lowest RR = 0.93. By contrast, the RR of CKD mortality increased over the years in the US, with its lowest in 1990–1994 (RR = 0.54) and its highest in 2015–2019 (RR = 1.65).

The cohort effect showed that the RR of CKD mortality in China increased in the cohort born between 1910 and 1914 (RR = 1.57) and the one born between 1945 and 1949 (RR = 2.21), then decreased to its lowest in the 2015–2019 cohort (RR = 0.11). The RR of CKD mortality in the US decreased with the progression of the cohort. The 1910–1914 cohort had the highest RR for CKD mortality (RR = 4.23), and the 2015–2019 cohort had the lowest RR for CKD mortality (RR = 0.13) (Fig. 2).

### Prediction of CKD, ASIR, and ASMR in China and the US

**Fig. 3:**
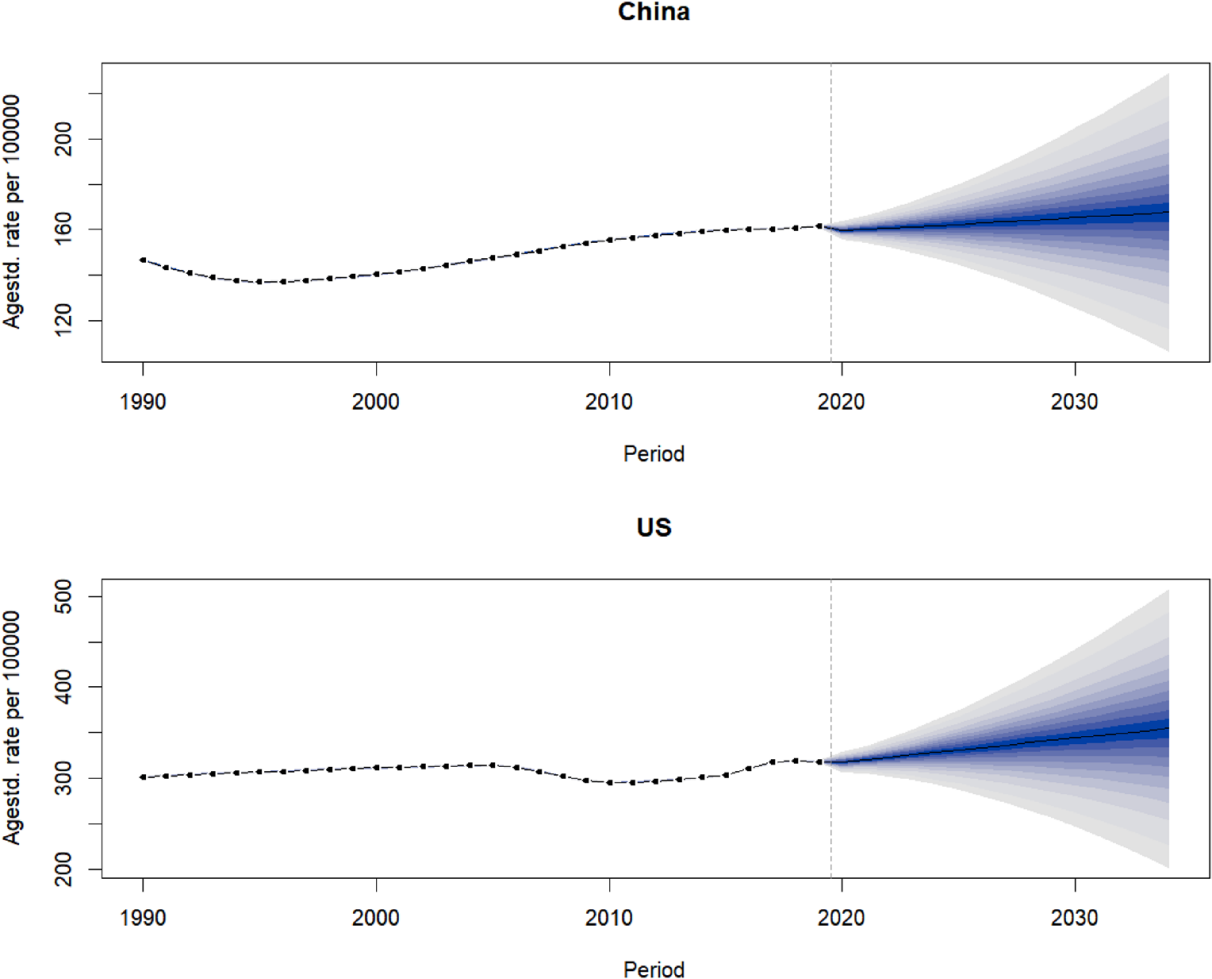
Prediction of CKD incidence in China and the US

**Fig. 4:**
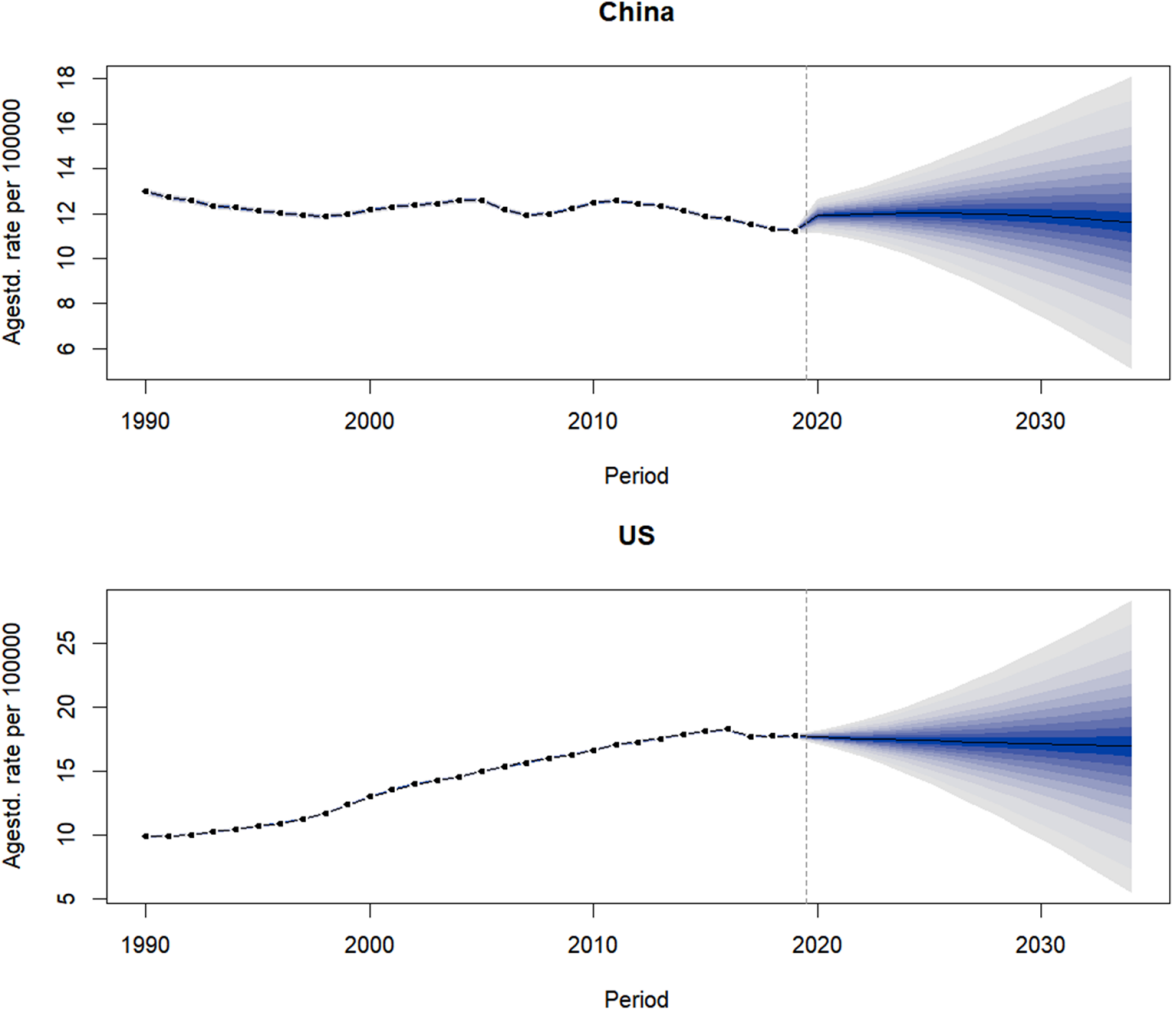
Prediction of CKD mortality in China and the US

Our prediction showed that the ASIR of CKD in China and the US will continue to rise over the next 15 years. We predicted that, by 2034, the ASIR of CKD in China will be 226.16/100,000, an increase of 4.99% compared to data from 2019. The ASIR of CKD in the US was 348.95/100,000, an increase of 11.48% compared to 2019 (Fig. 3). Our prediction showed that the CKD ASMR in China increased slightly in the first 5 years and then decreased. It is predicted that, by 2034, the ASMR of CKD in China will be 11.01/100,000, a decrease of 2.61% compared to data from 2019. The ASMR of CKD in the US will be 16.62/100,000, a decrease of 4.13% compared to 2019 (Fig. 4).

## Discussion

In this study, CKD incidence in China and the US is found to be increasing annually; the mortality rate of CKD in China is decreasing, and the mortality rate of CKD in the US is increasing. The RR of CKD incidence and mortality increased gradually with age in general; however, people aged < 5 years had a higher RR of CKD incidence than those in the other age categories. The RR of CKD incidence increased gradually over time, and the RR of CKD mortality in the US increased gradually as well. Later years of birth were associated with lower RRs of CKD incidence and mortality, and it is predicted that CKD incidence will increase, but the mortality rate will decline over the next 15 years.

Many factors affect the development of CKD, including age, sex, obesity, hypertension, diabetes mellitus, and hyperuricemia. The kidney is one of the target organs of obesity-related health disorders, a risk factor for CKD progression, and obesity is considered to be an independent risk factor for CKD development [18]. Studies have shown a U-shaped correlation between body mass index and renal function decline [19]. Diabetes is the most common cause of CKD worldwide. In the supplementary materials, a consistent upward trend in the ASIR of diabetes is observed in both China and the US, with the ASIR being consistently higher in the US. Additionally, the ASIR of CKD attributed to type 2 diabetes exhibits a comparable increasing trend in both countries, with the ASIR in the US exceeding that in China. These findings are consistent with the overarching results of the study, further substantiating the robust correlation between diabetes and CKD incidence. Compared to other causes of CKD, patients with CKD and diabetes are at a higher risk of developing end-stage renal disease (ESRD), which imposes a huge health and economic burden on both patients and society [20]. Hypertension is also a risk factor for CKD. Studies have shown that a systolic blood pressure (SBP) of 140 mmHg is associated with an increased risk of CKD incidence [21]. A decrease in SBP can significantly reduce mortality in patients with mild-to-moderate CKD and hypertension [22]. Studies have also shown that elevated serum uric acid is a risk factor for the development of both acute and chronic kidney diseases, as well as hypertension and diabetes. Uric acid may cause kidney disease by causing systemic and glomerular hypertension [23]. This study discovered that individuals aged 0–5 have an elevated RR of CKD incidence, with CKD in children resulting in significant height development and disproportionate growth disorders. Research has suggested that factors like hyperglycemia, vitamin A deficiency, and exposure to cocaine and alcohol during pregnancy can heighten the RR of CKD in infants [24]. Moreover, low birth weight and premature birth are also linked to an increased RR of CKD in infants [25]. The immature liver and kidney functions in children demand stringent dietary safety standards. Unhealthy dietary choices, such as inappropriate infant formula and adult food not suited for children, may harm their health, potentially leading to CKD. A notable example is China’s outbreak of pediatric urolithiasis due to melamine-contaminated milk powder. The Chinese Ministry of Health reported that approximately 294,000 children were diagnosed with urinary tract stones [26]. Studies have indicated that melamine-associated urolithiasis can cause damage to renal tubules and glomeruli [27]. This factor may contribute to the marginally higher RR of CKD among Chinese children aged 0–5 compared to their counterparts in the US.

The ASIR of CKD was found to increase in China and the US, and the period effect in the age-period-cohort analysis of CKD incidence also suggested that the RR of CKD incidence increased annually. With societal development over time, the aging of the population, and improvements to living standards, the risk factors for CKD also increase, and the incidence and risk of CKD also increase. In the 1990s, China’s economic level rose rapidly due to the implementation of the Economic Reform and Open Up approaches; the ASIR of the CKD changed from a downward trend to an upward one. The ASIR of CKD in the US decreased from 2005 to 2010, which may be attributed to the rapid growth in GDP and the improvement of health services in the US during this period. The ASIR for CKD was found to be higher in the US than in China. Research shows that in the US, 1/3 of the total population is obese, and another 1/3 is overweight. The obesity rate in the US is higher than that in China [18]. According to GBD data, the incidence of diabetes and hypertension in the US is higher than that in China, which may explain why the incidence of CKD in the US is higher.

Joinpoint Regression analysis suggested that the ASMR of CKD in China decreased, whereas that in the US increased. The period effect in the age-period-cohort analysis of CKD mortality suggested that the RR of CKD mortality in the US increased each year. In terms of age, the RR for CKD incidence and mortality in the US were higher than those in China. This may be because the Chinese government, which established the China Kidney Network (CK-NET) in 2014 [18], has continuously strengthened the reform of the medical system to ensure fairness of medical services for the masses, providing data to support the formulation of health policies. Other studies have pointed out that patients with CKD comprise a high proportion of African Americans and other ethnic and socially disadvantaged groups in the US, which also reflects the inequality of health service access and unfair health policy in the US [18]. This may explain the increased CKD mortality and higher RR of CKD mortality in the US.

The ASIR of CKD was found to be higher in women than in men, and the ASMR of CKD was higher in men than in women. The reason behind this difference remains unclear. It may be due to the fact that women generally have less muscle mass than men, and muscle mass is the main determinant of serum creatinine concentration, thus making CKD more detectable in women [28, 29]. Studies have shown that sex-based differences in systemic and renal hemodynamics and hypertension control, as well as differences in the effects of sex hormones on cell metabolism, lead to the slower progression of CKD in women than in men [6, 30]. This may explain why CKD mortality is higher in men than in women.

The cohort effect in our age-period-cohort analysis suggested that the RR of CKD incidence and mortality decreased with cohort size, which may be the result of the development of medical technologies over time. However, the RR of CKD mortality in Chinese people born between 1910 and 1949 is on the rise, which may be because China is in a period of war and turmoil, and the medical services of the population are not guaranteed, leading to an increase in the RR of mortality.

Our prediction shows that the incidence of CKD in China and the US will increase, and mortality will decline over the next 15 years. To enhance the prevention and treatment of CKD, both countries should proactively implement relevant health policies, particularly focusing on increasing healthcare accessibility in the US. This includes screening for high-risk CKD populations, notably those with hypertension and diabetes. CKD screening effectively delays CKD progression and reduces cardiovascular disease risk [31]. It is advisable to regularly monitor kidney function, urinary protein, blood pressure, and other indicators for early CKD detection and intervention. For patients with end-stage renal disease (ESRD), increasing access to dialysis and kidney transplantation is crucial to enhance their quality of life and prognosis. Given the higher obesity rate in the US compared to China, American residents need to adhere to a healthy diet to decrease obesity incidence, thereby reducing the risk of developing CKD. Regular physical activity and healthy eating habits are also important. Research has shown that adherence to a diet rich in whole grains, vegetables, fruits, beans, nuts, and fish, as well as reducing the intake of red meat and processed meat, sodium, and sugary drinks, are related to a lower RR of CKD incidence [32]. Physical exercise can effectively prevent the occurrence of CKD and improve both the quality of life and the blood pressure levels of patients with CKD [33].

The limitations of this study include potential inaccuracies in the GBD database data: while extensive, the data in GBD are prone to significant errors and rely heavily on estimations, potentially impacting the research findings. Additionally, as this study is ecological in nature, ecological fallacy cannot be avoided, with variations in population distribution and age differences possibly influencing the results. In this study, statistical models and years were employed to estimate the age-standardized incidence or mortality rates, yet the influences of economic, cultural, and health development levels, demographic shifts, or policy interventions on these rates were not considered. Furthermore, due to the absence of detailed data on the incidence and mortality of different CKD stages in the GBD database, this study is unable to provide an analysis of CKD at various stages.

## Conclusion

To improve the prevention and treatment of CKD in the future, we recommend enhancing CKD screening for high-risk groups and implementing relevant policies to ensure access to medical and healthcare services for older adults. Special attention should also be paid to the prenatal examination of pregnant women to reduce the occurrence of CKD in newborns. Health education programs that encourage individuals to adopt healthy dietary patterns, engage in regular physical exercise, and undergo routine medical checkups should be adopted. Furthermore, efforts should be made to strengthen tertiary prevention strategies for CKD and implement effective prevention and treatment measures.

### Electronic supplementary material

Below is the link to the electronic supplementary material.

Supplementary Material 1：CKD ASIR and ASMR

Supplementary Material 2：Age-period-cohort analysis of CKD incidence and mortality in China and the US

Supplementary Material 3：Prediction of CKD ASIR and ASMR

Supplementary Material 4：The ASIR of CKD attributed to diabetes and the ASIR of diabetes

## Data Availability

Data were obtained from the 2019 Global Burden of Disease (GBD)(https://ghdx.healthdata.org/gbd-2019).

## Abbreviations

AAPC: Average Annual Percentage Change
APC: Annual Percentage Change
ASIR: Age-Standardized Incidence Rate
ASMR: Age-Standardized Mortality Rate
CK-NET: China Kidney Network
CKD: Chronic Kidney Disease
ESRD: End-Stage Renal Disease
GBD: Global Burden of Disease
GFR: Glomerular Filtration Rate
IE: Intrinsic Estimator
RR: Relative Risk
SBP: Systolic Blood Pressure
SDI: Social Demographic Index

## References

[1] Stevens PE, Levin A, Kidney Disease, Improving Global Outcomes Chronic Kidney Disease Guideline Development Work Group Members (2013). Evaluation and management of chronic kidney disease: Synopsis of the kidney disease: improving global outcomes 2012 clinical practice guideline. Ann Intern Med.

[2] Vejakama P, Ingsathit A, Attia J, Thakkinstian A (2015). Epidemiological study of chronic kidney disease progression: a large-scale population-based cohort study. Med (Baltim).

[3] Jankowski J, Floege J, Fliser D, Böhm M, Marx N (2021). Cardiovascular disease in chronic kidney disease: pathophysiological insights and therapeutic options. Circulation.

[4] Chen TK, Knicely DH, Grams ME (2019). Chronic kidney disease diagnosis and management: a review. JAMA.

[5] GBD Chronic Kidney Disease Collaboration (2020). Global, regional, and national burden of chronic kidney disease, 1990–2017: a systematic analysis for the global burden of Disease Study 2017. Lancet.

[6] Swartling O, Rydell H, Stendahl M, Segelmark M, Trolle Lagerros Y, Evans M (2021). CKD progression and mortality among men and women: a nationwide study in Sweden. Am J Kidney Dis.

[7] GBD. (2015) Global, regional, and national age-sex specific all-cause and cause-specific mortality for 240 causes of death, 1990–2013: A systematic analysis for the Global Burden of Disease Study 2013. Lancet Jan 10;385(9963):117– 71 385(9963):117–171. 10.1016/S0140-6736(14)61682-2. 10.1016/S0140-6736(14)61682-2 PMC4340604 25530442

[8] Wang L, Xu X, Zhang M, Hu C, Zhang X, Li C (2023). Prevalence of chronic kidney disease in China: results from the sixth China chronic disease and risk factor surveillance. JAMA Intern Med.

[9] Murphy D, McCulloch CE, Lin F, Banerjee T, Bragg-Gresham JL, Eberhardt MS (2016). Trends in prevalence of chronic kidney disease in the United States. Ann Intern Med.

[10] Li Y, Ning Y, Shen B, Shi Y, Song N (2022). Temporal trends in prevalence and mortality for chronic kidney disease in China from 1990 to 2019: an analysis of the global burden of Disease Study 2019. Clin Kidney J.

[11] GBD (2016). Global, regional, and national disability-adjusted life-years (DALYs) for 315 diseases and injuries and healthy life expectancy (HALE), 1990–2015: a systematic analysis for the global burden of Disease Study 2015. Lancet.

[12] GBD 2019 Colorectal Cancer Collaborators. Global, regional, and national burden of colorectal cancer and its risk factors, 1990–2019: a systematic analysis for the Global Burden of Disease Study 2019. Lancet Gastroenterol Hepatol. 2022;7(7):627–647. doi: 10.1016/S2468-1253(22)00044-9. Epub 2022 Apr 7. Erratum in: Lancet Gastroenterol Hepatol. 2022;7(8):704. PMID: 35397795; PMCID: PMC9192760. 10.1016/S2468-1253(22)00044-9 PMC9192760 35397795

[13] Qiu H, Cao S, Xu R (2021). Cancer incidence, mortality, and burden in China: a time-trend analysis and comparison with the United States and United Kingdom based on the global epidemiological data released in 2020. Cancer Commun (Lond).

[14] Liu X, Jiang J, Yu C, Wang Y, Sun Y, Tang J (2019). Secular trends in incidence and mortality of bladder cancer in China, 1990–2017: a joinpoint and age-period-cohort analysis. Cancer Epidemiol.

[15] Yang Y, Schulhofer-Wohl S, Fu W, Land K (2008). The intrinsic estimator for age-period-cohort analysis: what it is and how to use it. Am J Sociol.

[16] Dong Z, Wang QQ, Yu SC, Huang F, Liu JJ (2022). Age-period-cohort analysis of pulmonary tuberculosis reported incidence, China, 2006–2020. Infect Dis Poverty.

[17] Yu J, Yang X, He W, Ye W (2021). Burden of pancreatic cancer along with attributable risk factors in Europe between 1990 and 2019, and projections until 2039. Int J Cancer.

[18] Alkaf B, Blakemore AI, Järvelin MR, Lessan N (2021). Secondary analyses of global datasets: do obesity and physical activity explain variation in diabetes risk across populations?. Int J Obes (Lond).

[19] Chintam K, Chang AR (2021). Strategies to treat obesity in patients with CKD. Am J Kidney Dis.

[20] Chen S, Chen L, Jiang H (2022). Prognosis and risk factors of chronic kidney disease progression in patients with diabetic kidney disease and non-diabetic kidney disease: a prospective cohort CKD-ROUTE study. Ren Fail.

[21] Mead D, Dinh N, Wentworth D, Thomas S, Suthar M (2021). Managing diabetes and hypertension in chronic kidney disease. Nurse Pract.

[22] Cheung AK, Rahman M, Reboussin DM, Craven TE, Greene T, Kimmel PL (2017). Effects of intensive BP control in CKD. J Am Soc Nephrol.

[23] Son YB, Yang JH, Kim MG, Jo SK, Cho WY, Oh SW (2021). The effect of baseline serum uric acid on chronic kidney disease in normotensive, normoglycemic, and non-obese individuals: a health checkup cohort study. PLoS ONE.

[24] Rosenblum S, Pal A, Reidy K (2017). Renal development in the fetus and premature infant. Semin Fetal Neonatal Med.

[25] Bach KE, Kelly JT, Palmer SC, Khalesi S, Strippoli GFM, Campbell KL (2019). Healthy dietary patterns and incidence of CKD: a meta-analysis of cohort studies. Clin J Am Soc Nephrol.

[26] Zhu SL, Li JH, Chen L, Bao ZX, Zhang LJ, Li JP et al. Conservative management of pediatric nephrolithiasis caused by melamine-contaminated milk powder. Pediatrics. 2009;123(6):e1099–e1102. 10.1542/peds.2008-3659. PMID: 19482743. 10.1542/peds.2008-3659 19482743

[27] Gao J, Wang F, Kuang X, Chen R, Rao J, Wang B, et al. Assessment of chronic renal injury from melamine-associated pediatric urolithiasis: an eighteen-month prospective cohort study. Ann Saudi Med. 2016 Jul-Aug;36(4):252–7. 10.5144/0256-4947.2016.252. PMID: 27478910; PMCID: PMC6074399. 10.5144/0256-4947.2016.252 PMC6074399 27478910

[28] Thurlow JS, Joshi M, Yan G, Norris KC, Agodoa LY, Yuan CM, Nee R (2021). Global epidemiology of end-stage kidney disease and disparities in kidney replacement therapy. Am J Nephrol.

[29] Hill NR, Fatoba ST, Oke JL, Hirst JA, O’Callaghan CA, Lasserson DS, Hobbs FD (2016). Global prevalence of chronic kidney disease– A systematic review and meta-analysis. PLoS ONE.

[30] Neugarten J, Golestaneh L (2019). Influence of sex on the progression of chronic kidney disease. Mayo Clin Proc.

[31] Kovesdy CP (2022). Epidemiology of chronic kidney disease: an update 2022. Kidney Int Suppl (2011) Apr.

[32] Nada A, Bonachea EM, Askenazi DJ (2017). Acute kidney injury in the fetus and neonate. Semin Fetal Neonatal Med.

[33] Shlipak, Tummalapalli, Boulware, Grams, Ix, Jha (2021). The case for early identification and intervention of chronic kidney disease: conclusions from a kidney disease: improving global outcomes (KDIGO) Controversies Conference. Kidney Int.

[34] Villanego F, Naranjo J, Vigara LA, Cazorla JM, Montero ME, García T (2020). Impact of physical exercise in patients with chronic kidney disease: sistematic review and meta-analysis. Nefrol (Engl Ed).

