# Supplementary material for: Trend analysis and prediction of the incidence and mortality of CKD in China and the US
Source: BMC Nephrol. 2024 Mar 1;25:76. doi: 10.1186/s12882-024-03518-w (PMC10908046; doi:10.1186/s12882-024-03518-w)
Supplement: Supplementary file 1 — Supplementary Material 1：CKD ASIR and ASMR [file 12882_2024_3518_MOESM1_ESM.pdf]

CKD ASIR and ASMR in China and the US(1/100,000)

Year	ASIR						ASMR					
	China	China:men	China:women	US	US:men	US:women	China	China:men	China:women	US	US:men	US:women
1990	146.38	141.33	152.72	301.57	283.88	316.39	12.98	14.82	12.07	9.94	12.55	8.45
1991	143.44	138.82	149.41	302.95	284.72	318.27	12.72	14.60	11.78	9.93	12.53	8.44
1992	140.91	136.70	146.51	304.29	285.62	320.05	12.57	14.43	11.63	10.00	12.63	8.50
1993	138.91	135.06	144.21	305.51	286.53	321.60	12.35	14.13	11.47	10.29	13.00	8.74
1994	137.57	134.00	142.66	306.53	287.39	322.79	12.27	14.03	11.40	10.48	13.17	8.93
1995	137.03	133.60	142.04	307.24	288.17	323.51	12.13	13.96	11.18	10.73	13.44	9.16
1996	137.17	133.82	142.15	308.01	289.12	324.17	12.03	13.87	11.06	10.96	13.60	9.43
1997	137.71	134.46	142.63	309.07	290.38	325.14	11.93	13.73	10.99	11.24	13.84	9.74
1998	138.52	135.36	143.36	310.21	291.71	326.19	11.87	13.75	10.87	11.69	14.29	10.17
1999	139.45	136.39	144.22	311.19	292.87	327.11	11.96	13.83	10.97	12.39	15.03	10.84
2000	140.38	137.37	145.09	311.79	293.63	327.66	12.15	13.96	11.17	13.01	15.65	11.46
2001	141.47	138.40	146.24	312.29	294.28	328.11	12.29	14.23	11.23	13.57	16.25	11.97
2002	142.91	139.62	147.88	312.98	295.14	328.76	12.38	14.46	11.23	14.01	16.84	12.29
2003	144.52	140.94	149.78	313.69	295.99	329.44	12.43	14.55	11.24	14.34	17.21	12.56
2004	146.14	142.27	151.69	314.25	296.61	330.00	12.60	14.88	11.28	14.53	17.33	12.79
2005	147.61	143.52	153.36	314.47	296.79	330.30	12.60	15.12	11.12	15.03	17.86	13.25
2006	149.10	144.83	155.01	312.54	294.85	328.29	12.15	14.57	10.77	15.37	18.11	13.63
2007	150.79	146.31	156.89	307.93	290.37	323.35	11.92	14.39	10.50	15.67	18.32	13.96
2008	152.51	147.81	158.82	302.40	285.02	317.44	11.98	14.56	10.49	16.02	18.81	14.20
2009	154.08	149.17	160.59	297.75	280.45	312.51	12.20	14.93	10.62	16.27	19.18	14.35
2010	155.32	150.24	162.01	295.73	278.33	310.55	12.49	15.36	10.83	16.60	19.70	14.52
2011	156.33	151.07	163.19	296.07	278.25	311.33	12.57	15.55	10.85	17.07	20.26	14.90
2012	157.31	151.85	164.39	297.20	278.72	313.13	12.45	15.73	10.58	17.27	20.59	14.99
2013	158.23	152.58	165.50	298.96	279.76	315.66	12.34	15.71	10.43	17.56	21.06	15.11
2014	159.01	153.25	166.39	301.21	281.37	318.58	12.14	15.46	10.22	17.85	21.36	15.38
2015	159.61	153.86	166.98	303.79	283.58	321.59	11.87	15.24	9.92	18.15	21.68	15.62
2016	159.85	154.50	166.89	310.94	291.04	328.38	11.74	15.05	9.81	18.29	21.92	15.66
2017	160.00	155.06	166.71	317.85	298.35	334.88	11.52	14.57	9.71	17.74	21.40	15.11
2018	160.58	155.67	167.25	319.32	300.18	336.33	11.33	14.11	9.65	17.74	21.64	14.93
2019	161.52	156.51	168.28	318.61	300.27	335.44	11.23	13.92	9.59	17.77	21.45	15.11
