# Supplementary material for: Trend analysis and prediction of the incidence and mortality of CKD in China and the US
Source: BMC Nephrol. 2024 Mar 1;25:76. doi: 10.1186/s12882-024-03518-w (PMC10908046; doi:10.1186/s12882-024-03518-w)
Supplement: Supplementary file 2 — Supplementary Material 2：Age-period-cohort analysis of CKD incidence and mortality in China and the US [file 12882_2024_3518_MOESM2_ESM.pdf]

Age-period-cohort analysis of CKD incidence and mortality in China and the US

	Incidence						Mortality					
	China			US			China			US		
	Coefficient	P	RR	Coefficient	P	RR	Coefficient	P	RR	Coefficient	P	RR
Age												
0～<5	0.69	0	1.99	0.36	0	1.44	-0.71	0	0.49	-0.70	0	0.50
5～<10	-1.48	0	0.23	-1.18	0	0.31	-1.66	0	0.19	-3.00	0	0.05
10～<15	-1.84	0	0.16	-1.71	0	0.18	-1.88	0	0.15	-3.13	0	0.04
15～<20	-1.77	0	0.17	-1.99	0	0.14	-1.40	0	0.25	-2.35	0	0.10
20～<25	-1.64	0	0.19	-2.14	0	0.12	-1.07	0	0.34	-1.67	0	0.19
25～<30	-1.48	0	0.23	-2.00	0	0.14	-1.00	0	0.37	-1.23	0	0.29
30～<35	-1.18	0	0.31	-1.68	0	0.19	-0.75	0	0.47	-0.81	0	0.45
35～<40	-0.73	0	0.48	-1.07	0	0.34	-0.54	0	0.58	-0.44	0	0.64
40～<45	-0.29	0	0.75	-0.32	0	0.72	-0.33	0	0.72	-0.08	0	0.92
45～<50	0.12	0	1.13	0.29	0	1.33	-0.17	0	0.84	0.29	0	1.33
50～<55	0.42	0	1.53	0.64	0	1.89	0.10	0	1.11	0.68	0	1.98
55～<60	0.72	0	2.05	1.03	0	2.79	0.42	0	1.52	1.08	0	2.94
60～<65	1.13	0	3.09	1.51	0	4.52	0.81	0	2.26	1.49	0	4.42
65～<70	1.54	0	4.68	1.83	0	6.23	1.26	0	3.52	1.85	0	6.38
70～<75	1.88	0	6.52	2.04	0	7.72	1.79	0	5.99	2.25	0	9.51
75～<80	1.98	0	7.25	2.20	0	9.06	2.33	0	10.28	2.67	0	14.39
80～<85	1.92	0	6.84	2.19	0	8.92	2.79	0	16.26	3.09	0	21.98
**Period**												
1990—1994	-0.30	0	0.74	-0.27	0	0.76	0.05	0.03	1.05	-0.62	0	0.54
1995—1999	-0.21	0	0.81	-0.16	0	0.86	-0.04	0.02	0.97	-0.34	0	0.71
2000—2004	-0.09	0	0.92	-0.05	0	0.95	0.05	0	1.05	-0.07	0	0.93
2005—2009	0.07	0	1.07	0.03	0	1.03	0.00	0.73	1.00	0.15	0	1.16
2010—2014	0.20	0	1.22	0.14	0	1.15	0.00	0.79	1.00	0.37	0	1.45
2015—2019	0.33	0	1.39	0.31	0	1.36	-0.07	0	0.93	0.50	0	1.65
**Cohort**												
1910—1914	0.92	0	2.51	1.14	0	3.13	0.45	0	1.57	1.44	0	4.23
1915—1919	0.90	0	2.46	1.05	0	2.86	0.51	0	1.66	1.34	0	3.82
1920—1924	0.85	0	2.35	0.97	0	2.63	0.58	0	1.79	1.22	0	3.39
1925—1929	0.79	0	2.20	0.89	0	2.44	0.69	0	1.99	1.09	0	2.99
1930—1934	0.70	0	2.02	0.79	0	2.21	0.75	0	2.11	0.96	0	2.61
1935—1939	0.61	0	1.84	0.67	0	1.94	0.76	0	2.14	0.84	0	2.30
1940—1944	0.50	0	1.66	0.55	0	1.74	0.77	0	2.16	0.70	0	2.01
1945—1949	0.39	0	1.48	0.45	0	1.56	0.79	0	2.21	0.59	0	1.80
1950—1954	0.29	0	1.33	0.34	0	1.40	0.74	0	2.09	0.48	0	1.62
1955—1959	0.19	0	1.21	0.23	0	1.26	0.62	0	1.86	0.40	0	1.49
1960—1964	0.10	0	1.10	0.12	0	1.13	0.54	0	1.71	0.28	0	1.32
1965—1969	0.00	0.87	1.00	-0.01	0.86	0.99	0.44	0	1.56	0.14	0	1.15
1970—1974	-0.10	0	0.90	-0.14	0.01	0.87	0.24	0	1.27	-0.01	0.81	0.99
1975—1979	-0.20	0	0.82	-0.28	0	0.76	0.08	0.29	1.09	-0.13	0	0.87
1980—1984	-0.30	0	0.74	-0.41	0	0.67	-0.08	0.37	0.93	-0.30	0	0.74
1985—1989	-0.43	0	0.65	-0.49	0	0.61	-0.26	0.01	0.77	-0.51	0	0.60
1990—1994	-0.56	0	0.57	-0.64	0	0.53	-0.46	0	0.63	-0.83	0	0.44
1995—1999	-0.66	0	0.52	-0.77	0	0.47	-0.66	0	0.52	-1.10	0	0.33
2000—2004	-0.74	0	0.48	-0.89	0	0.41	-1.05	0	0.35	-1.25	0	0.29
2005—2009	-0.86	0	0.42	-1.06	0	0.35	-1.45	0	0.23	-1.49	0	0.23
2010—2014	-1.04	0	0.35	-1.17	0	0.31	-1.81	0	0.16	-1.82	0	0.16
2015—2019	-1.37	0	0.25	-1.33	0	0.26	-2.20	0	0.11	-2.04	0	0.13
AIC	7.14			7.90			4.52			4.15		
BIC	-275.77			-246.75			-274.83			-277.28		
Deviance	1.73			30.75			2.66			0.22		
