# Supplementary material for: Trend analysis and prediction of the incidence and mortality of CKD in China and the US
Source: BMC Nephrol. 2024 Mar 1;25:76. doi: 10.1186/s12882-024-03518-w (PMC10908046; doi:10.1186/s12882-024-03518-w)
Supplement: Supplementary file 3 — Supplementary Material 3：Prediction of CKD ASIR and ASMR [file 12882_2024_3518_MOESM3_ESM.pdf]

Prediction of CKD ASIR and ASMR in China and the US(1/100,000)

Year	ASIR		ASMR	
	China	US	China	US
2020	159.57	318.12	11.90	17.68
2021	160.10	320.82	11.94	17.62
2022	160.61	323.51	11.97	17.56
2023	161.14	326.17	12.00	17.51
2024	161.68	328.82	12.01	17.46
2025	162.26	331.46	12.01	17.41
2026	162.82	334.12	12.01	17.36
2027	163.38	336.77	11.99	17.30
2028	163.95	339.38	11.96	17.25
2029	164.53	341.96	11.92	17.21
2030	165.14	344.54	11.88	17.16
2031	165.74	347.11	11.82	17.11
2032	166.34	349.66	11.75	17.06
2033	166.94	352.17	11.67	17.00
2034	167.54	354.63	11.59	16.95
